# Fetuin-B as a biomarker for metformin response in women with polycystic ovary syndrome: a prospective study

**DOI:** 10.3389/fmed.2025.1567255

**Published:** 2025-06-25

**Authors:** Konstantin Hofmann, Susanne Singer, Ann-Christin Meyer, Susanne Theis, Annette Hasenburg, Walburgis Brenner, Christine Skala

**Affiliations:** ^1^Department of Obstetrics and Gynecology, University Medical Center of Johannes Gutenberg University Mainz, Mainz, Germany; ^2^King's Fertility, Fetal Medicine Research Institute, London, United Kingdom; ^3^Institute of Medical Biostatistics, Epidemiology and Informatics, University Medical Center of Johannes Gutenberg University Mainz, Mainz, Germany

**Keywords:** PCOS, metformin, metabolism, fetuin-B, insulin resistance, obesity

## Abstract

**Introduction:**

Polycystic ovary syndrome (PCOS), affecting 5–15% of women of reproductive age globally, is linked to metabolic complications such as insulin resistance, obesity, and non-alcoholic fatty liver disease. While metformin helps manage PCOS, reliable biomarkers for monitoring treatment response are lacking. Fetuin-B, a liver-derived protein, has emerged as a potential candidate, as previous studies have shown increased fetuin-B levels in women with PCOS. This study examined whether serum fetuin-B levels correlated with metabolic improvements in PCOS patients undergoing metformin therapy, exploring its potential as a biomarker.

**Methods:**

PCOS patients from the Fertility Center at the University Medical Center Mainz were assigned to two groups: metformin therapy (M-group) and alternative/no treatment (C-group), based on their metabolic profiles. Baseline and 24-week follow-up assessments included gonadotropins and reproductive hormone levels, metabolic markers (such as lipid profile, hepatic markers, fasting and stimulated glucose levels, and the respective derived indices), and anthropometric data, including fetuin-B. A multivariate regression analysis evaluated associations between metabolic changes and fetuin-B levels.

**Results:**

A total of 62 PCOS patients were included (31 per group). At baseline, the M-group exhibited worse metabolic parameters compared to the C-group, including higher body mass index (BMI) (*p* < 0.001), Homeostatic Model Assessment for Insulin Resistance (HOMA-IR) (*p* < 0.001), waist circumference (*p* < 0.001), and fatty liver index (FLI) (*p* < 0.001). At follow-up, the M-group showed greater improvements. Fetuin-B levels were significantly higher in the M-group at baseline (*p* = 0.01), but at follow-up, no significant difference was observed between the groups (M-group: 3.7mcg/mL; C-group: 4.4 mcg/mL; *p* = 0.13). The M-group’s fetuin-B levels decreased significantly (*p* < 0.001), while the C-group’s levels increased slightly. Changes in fetuin-B levels differed significantly between groups (*p* < 0.001), and regression analysis confirmed a strong association (B: 1.80; 95% CIs: 0.57–3.03; *p* = 0.01).

**Conclusion:**

This study demonstrated that metformin therapy is associated with significantly reducing fetuin-B levels in PCOS patients, underscoring its role in enhancing metabolic health. These findings highlight fetuin-B as a potential biomarker for monitoring treatment efficacy, offering a link between metabolic and reproductive health.

## Introduction

Polycystic ovary syndrome (PCOS), first described in 1935, is a complex endocrine and metabolic disorder affecting approximately 5–15% of women aged 15–44 years globally, making it the most common endocrine disorder among premenopausal women (1–3). Beyond its detrimental impact on endocrine, psychological, and reproductive health, the metabolic aspect of PCOS plays a critical role in its clinical presentation. PCOS increases the likelihood of a range of metabolic disorders. For example, patients with PCOS may experience hyperinsulinemia, insulin resistance, obesity, non-alcoholic fatty liver disease (NAFLD), dyslipidemia, and metabolic syndrome, all of which elevate the risk of type II diabetes and other cardiovascular diseases (4–7). Insulin resistance, a hallmark of PCOS, not only contributes to the heightened risk of metabolic diseases but also complicates fertility treatment and affects the response to interventions such as *in vitro* fertilization (IVF) (8). Furthermore, metabolic abnormalities can exacerbate other clinical symptoms, including hirsutism, acne, and irregular menstrual cycles, thereby influencing a patient’s quality of life (2).

In the 2023 guideline for PCOS published by the European Society of Human Reproduction and Embryology (ESHRE), the role of pharmacological therapy in addressing the metabolic component of PCOS is outlined. If lifestyle modifications combined with oral contraceptives do not yield success, a pharmacological approach using biguanide metformin is recommended. For PCOS patients with metabolic disorders such as insulin resistance or obesity, metformin therapy may be initiated without prior use of oral contraceptives (2). Metformin’s role in improving insulin sensitivity and aiding weight loss has made it a commonly prescribed treatment for managing the metabolic aspects of PCOS (9). However, its long-term effects and the variability in individual responses highlight the need for more precise approaches to monitoring its efficacy and tailoring treatment strategies.

Despite these advancements in treatment strategies, a reliable biochemical marker to monitor the metabolic response to PCOS therapies remains elusive. While various serum markers have been explored, none have yet been validated for routine clinical use (10, 11). Fetuin-B is a plasma protein synthesized in the liver, classified within the cystatin family of cysteine protease inhibitors, and it plays a critical role in the fertilization of oocytes (12–14). Serum fetuin-B levels are increased in PCOS patients and have been linked to insulin resistance and NAFLD (15). In addition, higher fetuin-B levels have been observed in women with gestational diabetes, indicating its broader involvement in metabolic regulation (16).

While insulin resistance is a key factor in the metabolic disturbances observed in PCOS, the relationship between specific biomarkers and clinical outcomes remains poorly understood. Current markers, such as the Homeostatic Model Assessment for Insulin Resistance (HOMA-IR) and lipids, are useful in assessing metabolic status, but they do not offer the specificity required to evaluate treatment effects accurately. Given the significant metabolic burden associated with PCOS and the lack of reliable markers for monitoring treatment response, fetuin-B presents a promising candidate for evaluating the efficacy of pharmacological therapies targeting the metabolic component of PCOS. This study aimed to determine whether serum levels of fetuin-B correlate with metabolic improvements in PCOS patients undergoing metformin therapy and to assess its potential as a biomarker for therapeutic monitoring.

## Methods

### Study design and ethical considerations

This prospective cohort study involved the consecutive enrollment of participants, each undergoing comprehensive assessments. Follow-up evaluations were conducted 24 weeks after therapy initiation (t1) to measure treatment efficacy and its impact on metabolic parameters, which were assessed both at baseline (t0) and follow-up (t1).

The study was approved by the Ethics Committee of the Rhineland-Palatinate Medical Association (Landesärztekammer Rheinland-Pfalz, 2021-15680_1, 28.04.2021) and adhered to the principles of the “Declaration of Helsinki” (17). Informed consent was obtained from all participants involved in the study, with confirmation that they agreed to the publication of the collected data.

### Inclusion and exclusion criteria

Participants were enrolled from the Fertility Center at the University Medical Center Mainz. When necessary, additional participants were referred by gynecological specialists in Mainz or included through announcements on the German PCOS support group’s website and public postings across the city. The inclusion period spanned 10 months, from spring 2020 to winter 2021. Eligible participants could have initiated any PCOS-related therapy at enrollment, provided it was discontinued for 3 months prior to inclusion. Exclusion criteria included actively seeking pregnancy, age under 18 years, or a history of mental health or significant medical conditions affecting study variables. Written informed consent was mandatory. PCOS was diagnosed according to the ESHRE criteria, which are based on the Rotterdam consensus and define four phenotypes based on the presence of at least two out of the three features: ovulatory dysfunction, clinical and/or biochemical hyperandrogenism, and polycystic ovarian morphology (PCOM) (2).

### Diagnostics

Baseline assessments included measurements of hormone levels (anti-Müllerian hormone [electrochemiluminescence immunoassay (ECLIA)], androstenedione [liquid Chromatography with tandem mass spectrometry (LC–MS–MS)], dehydroepiandrosterone sulfate [ECLIA], estradiol [ECLIA], follicle-stimulating hormone [chemiluminescence microparticle immunoassay (CLIA)], luteinizing hormone [CLIA], progesterone [CLIA], prolactin [CLIA], sex hormone-binding globulin [CLIA], testosterone [CLIA], thyroid-stimulating hormone [CLIA], and 17-OH progesterone [LC–MS–MS]). Previous hormonal therapy, if administered, was discontinued for 3 months prior to the analysis. All hormone assessments were conducted during the follicular phase, specifically on cycle days 1 to 5. At t1, hormone levels, clinical chemistry, and CRP were reassessed, except for specific hormones such as anti-Müllerian hormone and OGTT. Body weight, height, and waist circumference (measured between the last rib and iliac crest) were recorded at each visit. BMI was categorized according to the WHO guidelines: ≤24.9 kg/m^2^ (healthy weight), 25–29.9 kg/m^2^ (overweight), and ≥30 kg/m^2^ (obesity) (18). Fetuin-B levels were measured in triplicate using the commercial ELISA kit “Human Fetuin-B DuoSet; R&D Systems, Minneapolis, USA” (R and D Systems Cat# DY1725, RRID: AB_3083709) in accordance with the manufacturer’s instructions.

#### Homeostatic Model Assessment for Insulin Resistance (HOMA-IR)

HOMA-IR was calculated as:

$\begin{array}{l} \text{HOMA}-\mathrm{IR}=\text{fasting insulin}\;(\mathrm{mU}/L)\;\\ \times \text{fasting glucose}\;(\text{nmol}/L)/22.5 \end{array}$

Values of >2 suggested insulin resistance (19).

#### Fatty liver index (FLI)

The fatty liver index (FLI) estimates the likelihood of hepatic steatosis using BMI, waist circumference, triglycerides (TG), and gamma-glutamyltransferase (GGT) (20).

$\begin{array}{l} \mathrm{FLI}=e^{x}/(1+e^{x})\times 100,\text{where}\;x=0.953\times \log_{e}(\mathrm{TG})+0.139\\ \times \mathrm{BMI}+0.718\times \log_{e}(\mathrm{GGT})+0.053\times (\text{waist circumference})\\ -15.745,\text{with}\;\mathrm{TG}\;\text{measured in mmol}/l,\mathrm{BMI}\;\text{in}\;\mathrm{kg}/m^{2},\\ \mathrm{GGT}\;\text{in}\;U/L,\text{and waist circumference in centimeter}. \end{array}$

#### Visceral adiposity index (VAI)

Although BMI is a standard measure of nutritional status, it does not reflect fat distribution. While magnetic resonance imaging and computed tomography are gold standards for assessing fat distribution, the visceral adiposity index (VAI) offers a simpler method to estimate cardiovascular risk based on BMI, waist circumference, triglycerides, and high-density lipoprotein cholesterol (HDL) (21, 22). The VAI is calculated using a gender-specific formula. However, a definitive pathological cutoff for VAI remains undefined (23, 24).

$\mathrm{VAI}=\text{waist circumference}(\mathrm{cm})/(\begin{array}{l} 36.58+(\mathrm{BMI}\times 1.89)\times \\ \left(\mathrm{TG}/0.81)\times (1.52/\mathrm{HDL}\right) \end{array})$

### Therapy

After diagnostic evaluation, guideline-recommended therapies were discussed with patients through shared decision-making. Metformin was initiated for those with insulin resistance (M-group), with monitoring of liver and kidney function throughout the study. The starting dose was 1,700 mg daily (850 mg twice daily), adjusted for tolerability or side effects such as bloating or diarrhea. Patients not receiving metformin (C-group) received individualized therapies, including oral contraceptives, progestin-only preparations, myo-inositol, or no treatment if medication was deemed unnecessary or declined. Therapy tolerability and side effects were documented. The study participants in the M-group were scheduled for follow-up appointments at 4 weeks and 12 weeks to monitor therapy tolerance and blood parameters.

### Statistical analysis

Data were analyzed using IBM SPSS Statistics Version 29, using descriptive and regression methods. Participant characteristics were summarized using means, standard deviations, and percentages. Group comparisons utilized the independent t-test for continuous variables and the χ^2^ test for categorical variables.

A multivariate linear regression analysis was conducted to evaluate the association between metabolic markers—such as changes in VAI (∆VAI), BMI (∆BMI), waist circumference (∆waist-circumference), FLI (∆FLI), LH/FSH ratio (ΔLH/FSH ratio), CRP (ΔCRP), HOMA-IR, and therapy group assignment (M-group or C-group)—and changes in fetuin-B over time (ΔFetuin-B). Potential confounders were selected based on clinically relevant factors, including age and PCOS phenotype. The final multivariate model was developed using a change-in-estimate approach, whereby variables were retained if their inclusion altered the regression coefficient (B) by more than 10% (25).

Prior to inclusion in the regression model, variables were screened for multicollinearity. Parameters with a Pearson’s or Spearman’s correlation coefficient greater than 0.7 were excluded to prevent simultaneous entry into the model. Associations were quantified using the regression coefficient (B), accompanied by corresponding *p*-values and 95% confidence intervals (CIs).

## Results

A total of 62 patients with a confirmed diagnosis of PCOS participated in the study. Of these 62 patients, 31 were prescribed metformin and included in the M-group, with an average daily dosage of 1,664.5 ± 254 mg. During treatment, 19% of participants reported persistent side effects after 24 weeks, including abdominal pain (10%), diarrhea (10%), and nausea (7%). No severe side effects were noted, and all liver and kidney function tests were negative for toxicity.

The remaining 31 patients, who did not receive metformin, comprised the C-group. Within this group, 14 patients (45%) used myo-inositol, 7 (23%) used a combined oral contraceptive, 2 (7%) used a progestin-only pill, 3 (10%) chose a levonorgestrel intrauterine device, and 5 (16%) did not receive any medication. Detailed information on the study cohort is provided in Tables 1, 2. No participants were withdrawn from the study prematurely. Both groups attended a second study visit after 24 weeks.

**Table 1:** Clinical data and hormonal measurements upon enrollment (t0) and at follow-up (t1) in both study groups (metformin versus comparison group).

Parameter	Overall	Metformin group	Comparison group	*p*	t1 Metformin Group (M1)	p (M0-M1)	t1 Comparison group (C1)	p (C0-C1)	∆M1-M0	∆C1-C0	p (∆M-group versus ∆C-group)
Age (years) Mean ± SD	26.1 ± 4.0	27.2 ± 3.9	26.7 ± 4.1	0.25	–	–	–	–	–	–	-
PCOS phenotype				0.33	–	–	–	–	–	–	-
A (hyperandrogenism + ovulatory dysfunction + PCOM) B (hyperandrogenism + ovulatory dysfunction) C (hyperandrogenism + PCOM) D (ovulatory dysfunction + PCOM)	66.1% (41) 14.5% (9) 16.1% (10) 3.2% (2)	64.5% (20) 12.9% (4) 22.6% (7) 0.0% (0)	67.7% (21) 16.1% (5) 9.7% (3) 6.5% (2)								
Anti-Müllerian hormone (AMH) (ng/mL) Mean ± SD	9.5 ± 5.5	8.8 ± 4.5	10.1 ± 6.4	0.37	–	–	–	–	–	–	-
Free Androgen Index (FAI) Mean ± SD	4.2 ± 2.4	4.8 ± 2.6	3.5 ± 2.1	**0.04**	4.6 ± 2.7	0.62	2.8 ± 1.8	0.05	−0.2 ± 1.7	−0.7 ± 2.0	0.35
Androstenedione (ng/mL) Mean ± SD	3.3 ± 1.6	3.5 ± 1.5	3.1 ± 1.6	0.37	2.9 ± 1.6	0.050	2.5 ± 1.2	**0.04**	−0.6 ± 1.6	−0.6 ± 1.4	0.89
Testosterone (ng/dL) Mean ± SD	52.7 ± 21.9	52.2 ± 16.8	53.1 ± 26.4	0.87	48.3 ± 15.7	0.29	47.6 ± 19.2	0.16	−3.9 ± 20.2	−5.5 ± 21.5	0.76
LH/FSH ratio Mean ± SD	1.6 ± 1.1	1.3 ± 0.8	1.8 ± 1.4	0.07	1.6 ± 1.0	0.18	1.5 ± 1.0	0.22	−0.3 ± 1.4	−0.3 ± 1.4	0.07

Values with *p* < 0.05 are shown in bold. C0: comparison group not taking metformin, at t0; C1: comparison group not taking metformin, at t1; FSH: follicle-stimulating hormone; LH: luteinizing hormone; M0: study group taking metformin, at t0; M1: study group taking metformin, at t1; PCOM: polycystic ovary morphology; PCOS: polycystic ovary syndrome; SD: standard deviation.

**Table 2:** Metabolic parameters and hepatic measurements upon enrollment (t0) and at follow-up (t1) in both study groups (metformin versus comparison group).

Parameter	Overall	Metformin-group	Comparison-group	*p*	t1 Metformin-Group (M1)	p (M0-M1)	t1 Comparison-Group (C1)	p (C0-C1)	∆M1-M0	∆C1-C0	p (∆M-group versus ∆C-group)
HOMA-IR Mean ± SD	2.2 ± 1.5	3.0 ± 0.6	1.3 ± 0.1	**<0.001**	–	–	–	–	–	–	–
Weight (kg) Mean ± SD	79.5 ± 22.2	89.0 ± 23.4	70.1 ± 16.4	**<0.001**	86.7 ± 23.9	**0.02**	70.4 ± 16.8	0.65	−2.3 ± 5.3	0.4 ± 4.4	**0.04**
BMI (kg/m^2^) Mean ± SD	28.4 ± 7.3	31.9 ± 7.3	24.8 ± 5.3	**<0.001**	31.1 ± 7.6	**0.02**	24.9 ± 5.3	0.72	−0.8 ± 1.9	0.1 ± 1.5	**0.04**
Waist circumference (cm) Mean ± SD	84.7 ± 16.9	92.6 ± 15.4	76.8 ± 14.5	**<0.001**	92.4 ± 16.4	0.87	76.9 ± 13.5	0.97	−0.2 ± 2.4	0.04 ± 6.5	0.89
Visceral Adiposity Index (VAI) Mean ± SD	3.4 ± 2.7	4.1 ± 2.4	2.7 ± 2.8	0.06	4.1 ± 2.3	0.99	4.0 ± 5.8	0.26	−0.004 ± 1.7	1.3 ± 6.0	0.28
Fatty Liver Index (FLI) Mean ± SD	33.8 ± 35.3	53.4 ± 35.5	14.2 ± 21.9	**<0.001**	4.3 ± 5.2	0.15	17.5 ± 23.5	**0.04**	−2.6 ± 9.4	3.3 ± 8.1	**0.01**
Alanine transaminase (ALT) (U/L) Mean ± SD	21.5 ± 9.1	22.9 ± 9.6	20.0 ± 8.4	0.22	22.0 ± 9.5	0.52	19.6 ± 9.2	0.86	−0.9 ± 7.1	0.4 ± 10.4	0.61
Aspartate transaminase (AST) (U/L) Mean ± SD	25.5 ± 8.1	25.9 ± 8.8	25.1 ± 7.5	0.72	23.3 ± 5.6	0.06	23.9 ± 3.6	0.54	−2.5 ± 6.9	−0.8 ± 7.2	0.38
Gamma-glutamyltransferase (GGT) (U/L) Mean ± SD	17.5 ± 7.7	20.0 ± 8.7	14.9 ± 5.7	**0.01**	17.3 ± 7.4	**0.01**	14.4 ± 6.9	0.66	−2.5 ± 5.0	−0.4 ± 4.2	0.07
C-reactive protein (CRP) (mg/L) Mean ± SD	3.4 ± 4.5	5.0 ± 5.2	1.9 ± 3.0	**0.01**	50.9 ± 35.1	0.26	1.9 ± 2.6	0.97	−0.7 ± 2.9	−0.01 ± 1.4	0.31

Values with *p* < 0.05 are shown in bold. BMI: body mass index; C0: comparison group not taking metformin, at t0; C1: comparison group not taking metformin, at t1; HOMA-IR: Homeostatic Model Assessment for Insulin Resistance; M0: study group taking metformin, at t0; M1: study group taking metformin, at t1; SD: standard deviation.

The M-group displayed significant metabolic disturbances, as shown by the analysis of clinical parameters such as weight, BMI, HOMA-IR, waist circumference, and FLI, while these features were absent in the C-group. The levels of hepatic transaminases and GGT in both groups were within the normal range at all time points. However, a significant improvement in GGT levels was observed in the M-group during treatment; however, no such change occurred in the C-group. Despite these metabolic differences, both groups were composed of patients diagnosed with PCOS. Importantly, the PCOS phenotypes were similar in both groups, indicating that the observed metabolic differences were not associated with variations in PCOS phenotypes.

The average time from the start of therapy to the follow-up visit was 31.1 ± 7.8 weeks for the M-group and 32.4 ± 10.7 weeks for the C-group. With metformin treatment, the metabolic situation in the M-group improved, as evidenced by significant reductions in weight, BMI, and FLI. These changes demonstrated notable differences in the metabolic parameters of the M-group. Relevant follow-up data are presented in Tables 1, 2.

**Figure 1:**
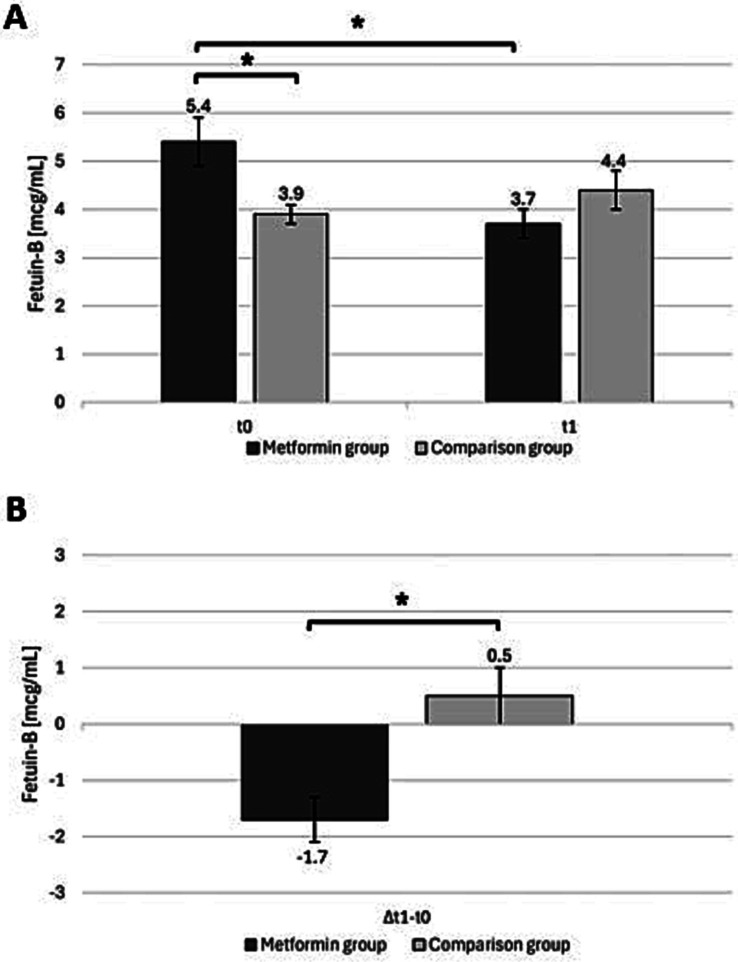
**(A)** Fetuin-B levels (mean scores ± standard errors) for patients with and without metformin, at study inclusion (t0) and follow-up (t1). **(B)** Change in fetuin-B levels (Δt1–t0) in both groups, shows the mean difference (mean scores ± standard errors) between baseline and follow-up. Significant differences between the time points are marked with an asterisk. Asterisks indicate statistical significance (*p* < 0.05).

At baseline (t0), the fetuin-B levels in the M-group were significantly higher than those in the C-group (*p* = 0.01). However, at follow-up (t1), no significant difference was observed between the groups, with the M-group receiving metformin therapy and the C-group receiving alternative treatments or no therapy (*p* = 0.13). The fetuin-B levels in the M-group decreased significantly (*p* < 0.001), while those in the C-group slightly increased. When examining the change (*Δ*) from t0 to t1 within each group, a significant difference between the groups was found (*p* < 0.001) (Figure 1).

### Regression analysis

Because of potential multicollinearity, Δweight was excluded from the model due to a correlation coefficient exceeding 0.7. The final model incorporated ΔVAI, Δwaist-circumference, group affiliation, ΔCRP, and HOMA-IR and was adjusted for age and PCOS phenotype, with ΔFetuin-B as the dependent variable.

The analysis revealed a significant association between group affiliation and ΔFetuin-B (B: 1.80; 95% CIs: 0.57–3.03; *p* = 0.01). The model demonstrated a goodness-of-fit with *R*^2^ = 0.15 and adjusted *R*^2^ = 0.13, based on a sample size of *N* = 50.

## Discussion

This study was the first to demonstrate that metformin therapy significantly reduced fetuin-B levels in PCOS patients, highlighting its potential to improve the metabolic profile in this population. At baseline, fetuin-B levels were significantly higher in the M-group compared to the C-group, but this difference was no longer observed at follow-up, as fetuin-B levels decreased significantly in the M-group while they showed a slight increase in the C-group. A multivariate regression analysis confirmed a significant association between group affiliation and changes in fetuin-B levels, indicating that metformin therapy played a key role in modulating this marker in PCOS patients (B: 1.80; 95% CIs: 0.57–3.03; *p* = 0.01). This observation aligned with the broader metabolic effects of metformin, including improvements in insulin sensitivity, lipid profiles, and markers of systemic inflammation (9). The reduction in fetuin-B levels further underscores its potential as a biomarker for monitoring metabolic responses in PCOS patients undergoing pharmacological treatment.

The 2023 guideline on PCOS recommends metformin as a pharmacological therapy for PCOS patients, particularly those with insulin resistance and overweight or obesity (2). However, monitoring the efficacy of metformin treatment and determining the appropriate duration of therapy remain challenging. Clinicians can face uncertainty concerning when to discontinue metformin, especially as its long-term use can be associated with gastrointestinal side effects such as diarrhea, abdominal pain, and nausea, which can negatively affect adherence (26). In this context, reliable biomarkers could play a critical role in assessing treatment response and guiding clinical decision-making. Unfortunately, no universally accepted biomarker has been established for monitoring therapeutic outcomes in PCOS, either for general management or in specific metabolic scenarios, leaving a significant gap in optimizing personalized care for these patients (27).

Fetuin-B levels are known to be elevated in PCOS patients and have been linked to adverse metabolic outcomes, including insulin resistance and increased risk of NAFLD (15, 28). Recent evidence revealed the significance of fetuin-B, a liver-derived glycoprotein, in the pathophysiology of PCOS and its possible modulation by metformin therapy. Beyond PCOS, elevated fetuin-B levels have also been linked to metabolic disorders, including type 2 diabetes, gestational diabetes, NAFLD, and insulin resistance, all of which are conditions that often co-occur with PCOS (15, 29, 30). These associations emphasize the dual role of fetuin-B as a biomarker and a potential mediator of metabolic dysfunction. By lowering fetuin-B levels, metformin could contribute to mitigating these risks, providing a supplementary therapeutic benefit for PCOS patients who are at heightened risk of metabolic disturbances.

In addition to its metabolic implications, fetuin-B plays a critical role in reproductive physiology. This protein is essential for the natural fertilization of oocytes, facilitating key interactions during sperm–egg fusion (31). Interestingly, increased fetuin-B levels have been associated with higher fertilization rates in IVF cycles (32, 33). Given that PCOS is frequently associated with subfertility, this finding raises intriguing questions about the dual impact of fetuin-B on metabolic and reproductive outcomes.

While reduced fertility rates are a hallmark of PCOS, the relationship between fetuin-B levels and fertility remains complex. The observed decrease in fetuin-B levels with metformin therapy, while beneficial for metabolic health, warrants further exploration concerning reproductive outcomes. Metformin is widely recognized as a fertility treatment in PCOS, as it can induce ovulation, regulate menstrual cycles, and improve overall reproductive potential (2). Understanding the balance between the metabolic and reproductive roles of fetuin-B could help refine therapeutic strategies, particularly for PCOS patients seeking to optimize fertility outcomes.

A study by Díaz et al. investigated the effects of a low-dose combination therapy consisting of spironolactone, pioglitazone, and metformin on fetuin-A levels in adolescent PCOS patients. Fetuin-A, a plasma glycoprotein structurally similar to fetuin-B, is known to play a role in metabolic regulation and is associated with the pathophysiology of diabetes mellitus (34). The study revealed that fetuin-A levels increased following combination therapy, despite being lower at baseline compared to a control group of PCOS patients treated with oral contraceptives. These findings are intriguing, as they contrast with other studies suggesting that fetuin-A levels are elevated in PCOS patients (35). The discrepancy highlights the complexity of fetuin-A dynamics in PCOS and the potential influence of therapeutic interventions (34).

In a study by Mokou et al., the impact of the GLP-1 receptor agonist liraglutide on fetuin-B levels was investigated over 6 months. The results demonstrated a significant reduction in fetuin-B levels with the therapy (28). However, it is important to note that the study had a small sample size and lacked a control group for comparison, which limits the generalizability of the findings. Furthermore, the therapeutic role of GLP-1 receptor agonists in the treatment of PCOS is still under investigation, as this medication is relatively new and its effects in PCOS patients are currently being explored.

Additionally, the anti-inflammatory properties of metformin, reflected in its ability to reduce CRP levels, may contribute to its therapeutic benefits. Although CRP levels declined in both study groups, the reduction was more substantial in the metformin-treated group, with average values remaining at the upper limit of the normal range. Given that elevated inflammatory markers are associated with conditions such as depression, anxiety, and cardiovascular disease, these findings underscore the broader significance of inflammation in the pathophysiology of PCOS (36–38).

The interplay between fetuin-B and metabolic health emphasizes its potential as a diagnostic and therapeutic target in PCOS. Interestingly, GGT levels declined in the metformin-treated group while remaining stable in the comparison group, mirroring the pattern observed for fetuin B. As GGT is one of the components of the fatty liver index (FLI)—a validated and more robust surrogate marker for hepatic steatosis than GGT alone—we incorporated FLI into our statistical analyses and regression models (20). The observed association supports the hypothesis that metformin’s therapeutic effects in PCOS may, at least in part, be mediated through improvements in hepatic metabolic function. The observed reduction in fetuin-B levels with metformin therapy offers a promising avenue for improving both metabolic outcomes in these patients. However, the reproductive implications of fetuin-B levels remain less clear and merit further investigation, particularly in light of its essential role in oocyte fertilization.

Future research should aim to clarify the mechanisms underlying fetuin-B modulation by metformin and its impact on fertility in PCOS. Moreover, prospective studies exploring the utility of fetuin-B as a biomarker for therapeutic monitoring and risk stratification in PCOS could help bridge the gap between metabolic and reproductive medicine.

### Strengths and limitations

Extensive diagnostic evaluations were conducted to accurately identify individuals with PCOS, enabling us to attribute the observed outcomes predominantly to characteristics specific to PCOS rather than unrecognized factors. Importantly, the FLI and VAI are not typically included in routine gynecological assessments, as they require additional laboratory tests, such as lipid profiles. Despite the critical role of metabolism in PCOS, research exploring the relationship between PCOS and key metabolic markers—including FLI, VAI, and HOMA-IR—remains limited.

The prospective cohort design allowed for a longitudinal evaluation of the impact of therapy over more than half a year, providing insights into both short- and long-term effects of metformin on metabolic parameters in PCOS patients. Nevertheless, a longer follow-up period could have provided more comprehensive data on the long-term effects of metformin therapy on fetuin-B and metabolic outcomes.

A larger sample size would have improved the reliability of our findings; however, enrollment challenges primarily contributed to the limited number of participants. In Germany, women with PCOS who are not undergoing fertility treatment are typically cared for in specialized gynecological practices rather than university clinics, which likely restricts the available participant pool. Additionally, relying on a university clinic for participant inclusion may have introduced selection bias, as patients in these settings often have more complex conditions. Those dissatisfied with prior treatments or experiencing higher levels of distress may have been more inclined to participate.

Our study used a comparison group rather than a true control group, which may limit the generalizability of our findings. Differences in the metabolic health status of study participants and the variety of treatments administered across groups could limit the comparability of results. Additionally, participants in the comparison group may not completely represent the clinical complexity or comorbidities typically observed in those with metabolic disorders, potentially introducing confounding variables. Furthermore, the variability in treatment approaches, which were not standardized across the comparison group, adds another layer of heterogeneity, making it more challenging to attribute observed effects to specific interventions. To mitigate these limitations, we performed a regression analysis after adjusting for several potential confounders, including markers of metabolic health. These adjustments aimed to account for baseline differences and increase the robustness of our findings, though residual confounding cannot be entirely ruled out. Future research should include a control group to enhance the robustness of such findings.

Sociocultural factors and ethnicity, which are known to influence metabolism, may have also contributed to bias, potentially affecting the measured metabolic outcomes (39). These factors, along with potential confounding by unmeasured variables—such as diet, physical activity levels, or genetic predisposition—complicated the interpretation of results and limited the ability to establish a clear causal relationship between metformin therapy, metabolism, and fetuin-B. This complication also reduced the applicability of the findings to diverse populations, as these unmeasured influences may vary across different groups.

## Conclusion

This study demonstrated that metformin therapy is associated with a significant reduction in fetuin-B levels in PCOS patients, underscoring its role in enhancing metabolic health. These findings highlight fetuin-B as a potential biomarker for monitoring treatment efficacy, offering a link between metabolic and reproductive health.

## Data Availability

The raw data supporting the conclusions of this article will be made available by the authors, without undue reservation.
